# Hermaphroditism with Adrenal Tumors

**Published:** 1913-02

**Authors:** 


Hermaphroditism With Adrenal Tumors.—Auvray, *Revue de Gyn. et de Chir. Abdom.*, reports a case of a woman aged 72, dead of intestinal obstruction. There was found a uterine myoma weighing 3½ pounds, pressing on the bowel at the pelvic brim. The ovaries were atrophic but showed traces of old corpora lutea. The clitoris was much enlarged. The left suprarenal contained an adeno-angeio-lipoma and was of the size of a cocoa-nut. Eight similar cases were found in the literature. Glynn and Apert hold that hyperplastic tumors of the adrenal, occurring before birth, leave the uterus above the cervix and the ovaries normal, but tend to the development of pseudo-hermaphroditism. Occurring in childhood, they tend to produce precocious menstruation and sexual maturity. Late in life they tend to produce an early menopause with obesity and hirsuties.

